# MRGBP is a potential novel prognostic biomarker and is correlated with immune infiltrates in hepatocellular carcinoma

**DOI:** 10.1097/MD.0000000000025234

**Published:** 2021-03-26

**Authors:** Juanjun Huang, Xiaoli Chen, Wei Zhu

**Affiliations:** a Department of Infectious Diseases; b Central Laboratory, the Affiliated Ganzhou Hospital of Nanchang University, Ganzhou, Jiangxi, PR China.

**Keywords:** biomarker, hepatocellular carcinoma, immune infiltrates, MORF4-related gene-binding protein, prognosis

## Abstract

This study investigated the expression change, prognostic values, and potential regulatory mechanisms of mortality factor on chromosome 4 (MORF4)-related gene-binding protein (MRGBP) in hepatocellular carcinoma (HCC).

MRGBP expression and clinical data from The Cancer Genome Atlas were used to evaluate the associations between MRGBP expression and clinicopathological characteristics. Kaplan–Meier and Cox regression analyses were performed to assess the factors contributing to prognosis. Gene set enrichment analysis (GSEA) was used to identify pathways associated with MRGBP expression. Single-sample gene set enrichment analysis (ssGSEA) was used to comprehensively analyze the relative immune infiltration levels.

High MRGBP expression was significantly associated with a higher T stage, pathologic stage, histologic grade, vascular invasion, tumor protein p53 status, and worse overall survival. MRGBP exhibited high diagnostic accuracy with an area under the receiver operating characteristic curve value of 0.980. GSEA revealed the enrichment of pathways related to tumorigenesis in the MRGBP high-expression phenotype, such as cell cycle and DNA replication pathways. ssGSEA revealed that MRGBP expression was significantly correlated with 15 types of immune cell infiltration levels. The Wilcoxon rank sum test revealed significantly high T helper (Th), T follicular helper, CD56 bright natural killer, and Th2 cell enrichment scores in the high MRGBP expression group and significantly low neutrophil, Th17, dendritic cell (DC), gamma delta T, cytotoxic cell, regulatory T cell, plasmacytoid DC, and immature DC enrichment scores.

MRGBP may be a novel prognostic biomarker and a therapeutic target correlated with immune infiltrates in HCC.

## Abstract 2

Supplemental Digital Content is available in the text

## 1 Introduction

Hepatocellular carcinoma (HCC) comprises 75% to 85% of primary liver carcinoma cases. HCC was the sixth most common cancer (fifth for males) and the fourth most common cause of death (second for males) worldwide in 2018. By 2040, estimates are for 1.35 million new cases and 1.28 million HCC-related deaths annually.^[1,2]^ Approximately 10% of patients with HCC show metastases at the time of diagnosis.^[3]^ Treatments, including surgical resection, transplantation, ablation, transarterial chemoembolization, and sorafenib, have improved patient survival. With the development of molecular targeted therapies, identifying novel targets and prognostic predictors through molecular profiling could further improve survival.^[4]^

Mortality factor on chromosome 4 (MORF4)-related gene-binding protein (MRGBP), also known as chromosome 20 open reading frame 20, encodes a subunit of the tat-interacting protein 60/histone acetyltransferase (TIP60/HAT) complex. The protein binds directly to 2 basic components of the TIP60/HAT complex and histone deacetylase complexes: MORF4-related gene on chromosome 15 and MORF4-related gene on chromosome X proteins.^[5]^ MRGBP is frequently amplified in numerous types of cancer, including lung,^[6]^ prostate,^[7,8]^ and pancreatic cancers^[9,10]^; cutaneous squamous cell carcinoma^[11]^; and colorectal^[12–14]^ and cervical cancers,^[15]^ and is involved in the regulation of the cell cycle, apoptosis, growth, and invasion.^[8,11,13,15]^ MRGBP may play a biological role as a diagnostic biomarker and anticancer target for tumors. However, little is known about the relationship between MRGBP and HCC.

In this study, we demonstrate for the first time the relationship between MRGBP and HCC, prognostically relevant expression profiles, and the correlation using bioinformatics analysis between immune infiltrates and MRGBP expression. The findings could provide new and promising insights for subsequent research to elucidate the clinicopathological significance and molecular pathogenesis of HCC.

## 2 Methods

### 2.1 RNA-sequencing (RNASeq) and clinical information

We evaluated the gene expression of 421 liver HCC samples comprising 371 tumor samples and 50 normal paracancer samples from the UCSC Xena database (https://xenabrowser.net/datapages/**)** using RNASeq (HTSeq-Counts). The clinical data of the corresponding patients were obtained from The Cancer Genome Atlas (TCGA) website **(**https://portal.gdc.cancer.gov/**)**. We obtained matched prognostic data from an Integrated TCGA Pan-Cancer Clinical Data Resource.^[16]^ HTSeq-counts and clinical data of 371 patients were extracted for further analysis **(**Table 1**)**. The 371 patients were divided into high and low groups according to the median MRGBP expression in tumor samples. As all the data used were retrieved from these online databases, there were no ethical issues.

**Table 1:** Demographic and clinical characteristics based on gene expression in TCGA cohort.

		Low expression of MRGBP	High expression of MRGBP	
Characters	Level	186	185	*P* value
T stage (%)	T1	104 (56.8%)	77 (41.6%)	.032^∗^^,^^†^
	T2	38 (20.8%)	56 (30.3%)	
	T3	35 (19.1%)	45 (24.3%)	
	T4	6 (3.3%)	7 (3.8%)	
N stage (%)	N0	125 (99.2%)	127 (97.7%)	.622^‡^
	N1	1 (0.8%)	3 (2.3%)	
M stage (%)	M0	130 (98.5%)	136 (98.6%)	1^‡^
	M1	2 (1.5%)	2 (1.4%)	
Pathologic stage (%)	Stage I	95 (55.2%)	76 (43.4%)	.14^‡^
	Stage II	38 (22.1%)	48 (27.4%)	
	Stage III	36 (20.9%)	49 (28.0%)	
	Stage IV	3 (1.7%)	2 (1.1%)	
Residual tumor (%)	R0	169 (97.1%)	155 (92.3%)	.025^∗^^,^^‡^
	R1	4 (2.3%)	13 (7.7%)	
	R2	1 (0.6%)	0 (0.0%)	
Histologic grade (%)	G1	41 (22.3%)	14 (7.7%)	<.001^∗^^,^^†^
	G2	104 (56.5%)	73 (40.1%)	
	G3	35 (19.0%)	87 (47.8%)	
	G4	4 (2.2%)	8 (4.4%)	
Gender (%)	Female	57 (30.6%)	64 (34.6%)	.484^†^
	Male	129 (69.4%)	121 (65.4%)	
Race (%)	Asian	70 (39.1%)	88 (48.9%)	.174^†^
	Black or African American	9 (5.0%)	8 (4.4%)	
	White	100 (55.9%)	84 (46.7%)	
Adjacent hepatic tissue inflammation (%)	Mild	48 (36.4%)	51 (50.0%)	.073^†^
	None	71 (53.8%)	46 (45.1%)	
	Severe	13 (9.8%)	5 (4.9%)	
Child-Pugh grade (%)	A	114 (89.1%)	103 (92.8%)	.566^‡^
	B	13 (10.2%)	8 (7.2%)	
	C	1 (0.8%)	0 (0.0%)	
Fibrosis ishak score (%)	0	46 (37.4%)	28 (31.5%)	.274^†^
	1–2	14 (11.4%)	17 (19.1%)	
	3–4	14 (11.4%)	14 (15.7%)	
	5–6	49 (39.8%)	30 (33.7%)	
Vascular invasion (%)	No	116 (70.7%)	90 (59.6%)	.05^†^
	Yes	48 (29.3%)	61 (40.4%)	
Tumor status (%)	Tumor free	110 (62.1%)	91 (52.0%)	.069^†^
	With tumor	67 (37.9%)	84 (48.0%)	
TP53 status (%)	Mut	31 (17.1%)	71 (40.1%)	<.001^∗^^,^^†^
	WT	150 (82.9%)	106 (59.9%)	
Age (median [IQR])		61.00 [53.00,68.75]	61.00[51.00,69.00]	.564^§^
Height (median [IQR])		168.00[161.00,175.00]	166.00[160.75,172.00]	.066^§^
Weight (median [IQR])		74.00 [61.00,88.00]	67.00[58.00,76.00]	.001^∗^^,^^§^
BMI (median [IQR])		25.35 [22.18,30.11]	23.88[21.45,27.14]	.005^∗^^,^^§^
AFP (ng/mL) (median [IQR])		7.00 [3.00,33.00]	52.50[7.00,2495.25]	<.001^∗^^,^^§^
Albumin (g/dL) (median [IQR])		3.90 [3.30,4.30]	4.10[3.60,4.30]	.127^§^
Prothrombin time (median [IQR])		1.10 [1.00,10.17]	1.00[1.00,1.30]	.001^∗^^,^^§^

AFP = alpha-fetoprotein, BMI = body mass index, IQR = interquartile range, MRGBP = MORF4-related gene-binding protein, TCGA = the cancer genome atlas, TP53 = tumor protein p53.

∗ Statistically significant.

† *χ*^2^ test.

‡ Fisher exact test.

§ Wilcoxon rank sum test.

### 2.2 Gene set enrichment analysis (GSEA)

GSEA was performed using R package clusterprofiler (3.6.0) to elucidate the potentially significant pathways associated with differentially expressed proteins in the high- and low-MRGBP groups. To identify the significantly enriched pathways, the number of permutations was 1000. The pathway sets with an adjusted *P* value < .05, false discovery rate (FDR) q-value < 0.25, and a |normalized enrichment score (NES) | > 1 were identified as significantly enriched.

### 2.3 Immune infiltration analysis using single-sample GSEA (ssGSEA)

The ssGSEA method from the Gene Set Variation Analysis package **(**http://www.bioconductor.org/packages/release/bioc/html/GSVA.html**)** in R (v 3.6.3) was used to comprehensively analyze the relative tumor cell infiltration levels, based on the signature gene lists of 24 types of immune cells.^[17]^ Spearman correlation was used to analyze the correlation between MRGBP and immunocytes. The Wilcoxon rank sum test was used to determine the immune infiltration differences among the different expression groups of MRGBP.

### 2.4 Statistical analyses

Statistical analyses were performed using R software (v 3.6.3). *χ*^2^ test, Wilcoxon rank sum test, and univariate logistic regression were performed to evaluate the association between MRGBP expression and the clinicopathological characteristics of patients. Survival curves were plotted using the Kaplan–Meier method and compared using the log-rank test. Survival data were evaluated using univariate and multivariate Cox regression analyses. Bivariate correlations between study variables were calculated using Spearman rank correlation coefficient. A *P* value < .05 was considered statistically significant in all tests.

## 3 Results

### 3.1 Demographic characteristics

TCGA data of 371 patients included their characteristics regarding the T, N, M, and pathologic stages, residual tumor, histologic grade, sex, race, adjacent hepatic tissue inflammation, Child–Pugh grade, fibrosis Ishak score, vascular invasion, tumor status, tumor protein p53 (TP53) status, age, height, weight, body mass index (BMI), alpha-fetoprotein (AFP), albumin, and prothrombin time. *X*^*2*^ analysis showed that MRGBP expression was significantly associated with the T stage (*P* = .032), residual tumor (*P* = .025), histologic grade (*P* < .001), and TP53 status (*P* < .001). The results of the Wilcoxon rank sum test showed that MRGBP expression was significantly associated with weight (*P* = .001), BMI (*P* = .005), AFP (*P* < .001), and prothrombin time (*P* = .001) (Table 1).

### 3.2 Associations between gene expression and clinicopathological features

**Figure 1:**
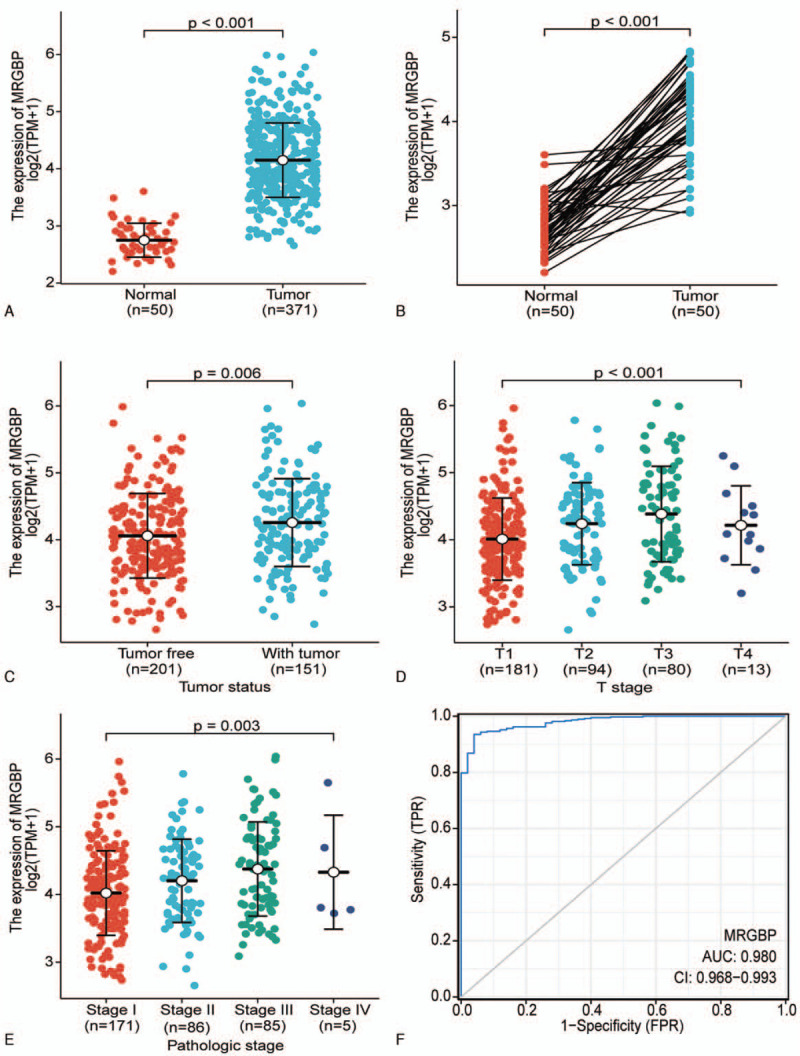
Differential expression of MRGBP and its association with clinicopathologic characteristics in TCGA. A, Normal tissues (n = 50) versus tumor tissues (n = 371). B, Normal tissues (n = 50) versus paired tumor tissues (n = 50). C–E, Associations between the MRGBP expression and tumor status, T stage, pathologic stage. F, ROC curves of MRGBP expression to predict HCC. FPR = false positive rate, HCC = hepatocellular carcinoma, MRGBP = MORF4-related gene-binding protein, ROC = receiver operating characteristic, TCGA = The Cancer Genome Atlas, TPM = transcripts per million, TPR = true positive rate.

Using the Wilcoxon signed-rank test, we found that the expression levels of MRGBP in 371 tumor tissues were notably higher than those in 50 normal tissues (*P* < .001; Fig. 1A). The values of MRGBP expression in 50 tumor tissues were remarkably higher than those in 50 paired normal liver tissues in TCGA cohort (*P* < .001; Fig. 1B). The higher expression of MRGBP correlated significantly with poor tumor status (*P* = .006), a higher T stage (*P* < .001), and a higher pathologic stage (*P* = .003) (Fig. 1, C–E**)**. In addition, MRGBP exhibited high diagnostic accuracy with an area under the receiver operating characteristic curve value of 0.980 **(**Fig. 1F**)**.

Univariate logistic regression analysis showed that high MRGBP expression was significantly associated with poor prognostic characteristics, including a higher T stage (odds ratio [OR] = 1.85 for T2, T3, and T4 vs T1, *P* = .004), pathologic stage (OR = 1.61 for Stages II, III, and IV vs Stage I, *P* = .028), histologic grade (OR = 4.06 for G3 and G4 vs G1 and G2, *P* < .001), vascular invasion (OR = 1.64 for Yes vs No, *P* = .039), and TP53 status (OR = 3.24 for Mut vs WT, *P* < .001) (Table 2). These results suggested that HCC with a higher MRGBP expression may progress to a poorer stage and vascular invasion.

**Table 2:** Association between gene expression and clinicopathologic features (logistic regression).

Characteristics	Total (N)	(OR) in MRGBP expression	*P* value
T stage (T2 and T3 and T4 vs T1)	368	1.85 (1.22–2.80)	.004^∗^
N stage (N1 vs N0)	256	2.95 (0.37–60.13)	.351
M stage (M1 vs M0)	270	0.96 (0.11–8.06)	.964
Pathologic stage (Stage II and Stage III and Stage IV vs Stage I)	347	1.61 (1.05–2.46)	.028^∗^
Histologic grade (G3 and G4 vs G1 and G2)	366	4.06 (2.59–6.47)	<.001^∗^
Residual tumor (R1 and R2 vs R0)	342	2.83 (1.04–9.00)	.053
Child-Pugh grade (B and C vs A)	239	0.63 (0.24–1.54)	.323
Fibrosis ishak score (1/2 and 3/4 and 5/6 vs 0)	212	1.30 (0.73–2.33)	.371
Adjacent hepatic tissue inflammation (mild and severe vs none)	234	1.42 (0.84–2.39)	.188
Vascular invasion (yes vs no)	315	1.64 (1.03–2.62)	.039^∗^
Tumor status (with tumor vs tumor free)	352	1.52 (0.99–2.32)	.055
TP53 status (Mut vs WT)	358	3.24 (2.00–5.34)	<.001^∗^

MRGBP = MORF4-related gene-binding protein, Mut = mutant type, OR = odds ratio, TP53 = tumor protein p53, WT = wild-type.

∗ Statistically significant.

### 3.3 Survival outcomes and Cox regression analysis

**Figure 2:**
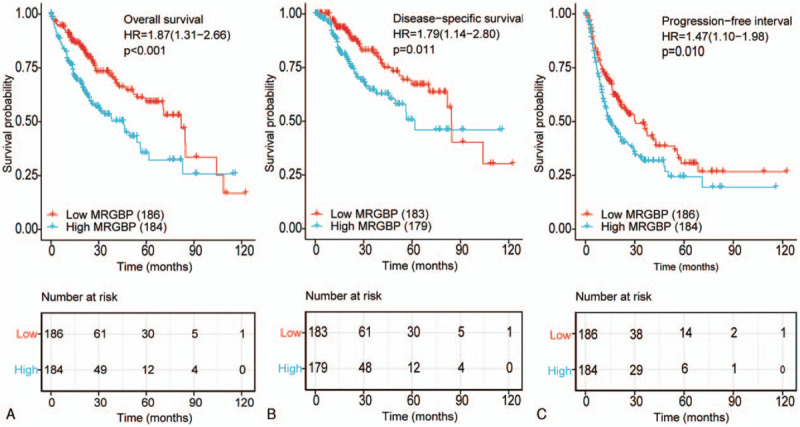
Kaplan–Meier survival analysis of HCC patients regarding MRGBP expression. A, Overall survival. B, Disease-specific survival. C, Progression-free interval. HCC = hepatocellular carcinoma, HR  =  hazard ratio, MRGBP = MORF4-related gene-binding protein.

Kaplan–Meier survival analysis indicated that HCC with a high expression of MRGBP had a worse overall survival (OS) (hazard ratio [HR] = 1.87 [1.31–2.66], *P* < .001), progression-free interval (HR = 1.47 [1.10–1.98], *P* = .010), and disease-specific survival (HR = 1.79 [1.14–2.80], *P* = .011) than HCC with low MRGBP expression (Fig. 2**)**.

Univariate analysis showed that a high MRGBP expression was significantly correlated with a worse OS (HR = 1.869 [1.315–2.655]; *P* < .001). Other clinicopathologic variables, including T, M, and pathologic stage and tumor status, were also associated with poor survival. In a multivariate analysis, high MRGBP expression remained independently associated with a poor OS (HR = 1.737 [1.061–2.845]; *P* = .028), along with the tumor status (Table 3).

**Table 3:** Univariate and multivariate analyses of various prognostic parameters and OS in patients with HCC (*cox-regression analysis*).

Characteristics	Total (N)	HR (95% CI) Univariate analysis	*P* value	HR (95% CI) Multivariate analysis	*P* value
T stage (T2 and T3 and T4 vs T1)	367	2.109 (1.469–3.028)	<.001^∗^	0.906 (0.122–6.745)	.923
N stage (N1 vs N0)	256	2.004 (0.491–8.181)	.333		
M stage (M1 vs M0)	270	4.032 (1.267–12.831)	.018^∗^	1.653 (0.393–6.949)	.493
Pathologic stage (Stage II and Stage III and Stage IV vs Stage I)	346	2.074 (1.418–3.032)	<.001^∗^	2.493 (0.324–19.169)	.380
Histologic grade (G3 and G4 vs G1 and G2)	365	1.120 (0.781–1.606)	.539		
Residual tumor (R1 and R2 vs R0)	341	1.571 (0.795–3.104)	.194		
Age (>60 vs <=60)	370	1.248 (0.880–1.768)	.214		
Gender (male vs female)	370	0.816 (0.573–1.163)	.260		
Weight (>70 vs <=70)	343	0.916 (0.640–1.312)	.634		
Height (>=170 vs < 170)	338	1.208 (0.833–1.753)	.319		
BMI (>25 vs <=25)	334	0.818 (0.563–1.186)	.289		
Race (White vs Asian and Black or African American)	358	1.245 (0.867–1.789)	.235		
Child-Pugh grade (B and C vs A)	238	1.616 (0.797–3.275)	.183		
AFP (ng/mL) (>400 vs <=400)	277	1.056 (0.646–1.727)	.827		
Albumin (g/dL) (>=3.5 vs <3.5)	296	0.921 (0.565–1.503)	.743		
Prothrombin time (>4 vs <=4)	293	1.330 (0.877–2.015)	.179		
Fibrosis ishak score (1/2 and 3/4 and 5/6 vs 0)	211	0.779 (0.470–1.293)	.334		
Adjacent hepatic tissue inflammation (mild and severe vs none)	233	1.228 (0.755–1.997)	.409		
Vascular invasion (yes vs no)	314	1.348 (0.890–2.042)	.159		
Tumor status (with tumor vs tumor free)	351	2.361 (1.620–3.441)	<.001^∗^	2.323 (1.415–3.815)	<.001^∗^
TP53 status (Mut vs WT)	357	1.434 (0.972–2.115)	.069	1.369 (0.806–2.325)	.245
MRGBP (high vs low)	370	1.869 (1.315–2.655)	<.001^∗^	1.737 (1.061–2.845)	.028^∗^

AFP = alpha-fetoprotein, BMI = body mass index, CI = confidence interval, HCC = hepatocellular carcinoma, HR = hazard ratio, MRGBP = MORF4-related gene-binding protein, Mut = mutant type, OS = Overall Survival, TP53 = tumor protein p53, WT = wild-type.

∗ Statistically significant.

### 3.4 GSEA identification of MRGBP-related Kyoto Encyclopedia of Genes and Genomes (KEGG) pathways

**Figure 3:**
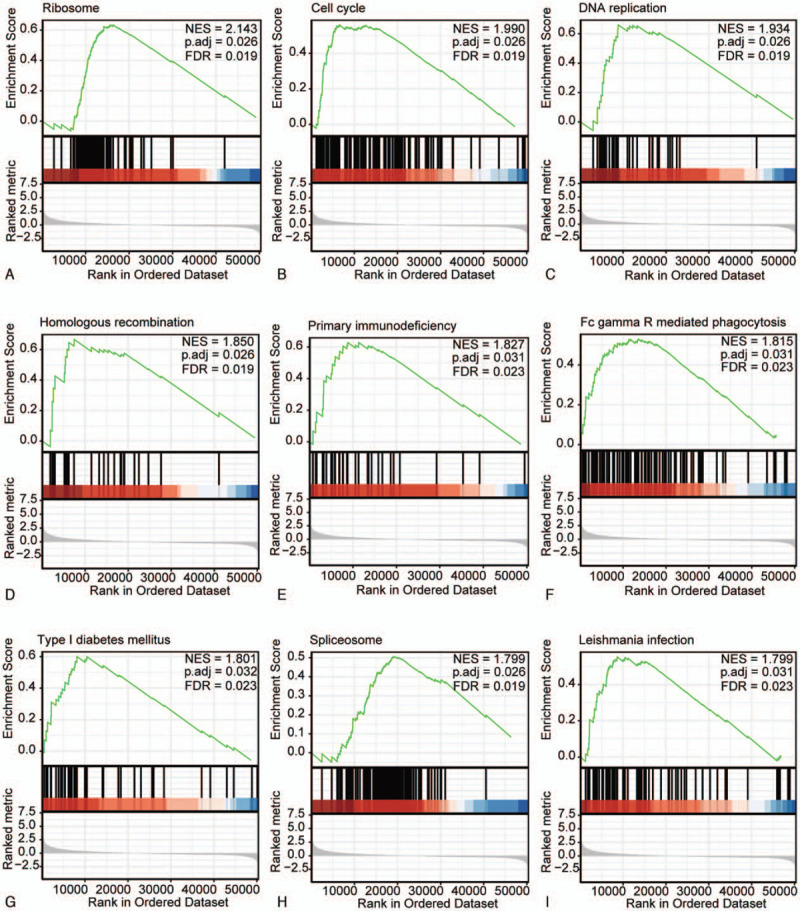
KEGG pathway enrichment analysis of MRGBP. Enrichment of genes in the KEGG ribosome (A), cell cycle (B), DNA replication (C), homologous recombination (D), primary immunodeficiency (E), Fc gamma R-mediated phagocytosis (F), type I diabetes mellitus (G), spliceosome (H), and leishmania infection (I) pathways using GSEA. FDR = false discovery rate, GSEA = gene set enrichment analysis, KEGG = Kyoto Encyclopedia of Genes and Genomes, MRGBP = MORF4-related gene-binding protein, NES = normalized enrichment score, TCGA = The Cancer Genome Atlas.

To identify different activated signaling pathways in HCC, GSEA was performed between MRGBP low-expression and high-expression datasets, with significant enrichment differences (FDR q < 0.05, *P* < .05, |NES| > 1) using the molecular signatures database collection (C2.cp.v7.0.symbols.gmt). Sixty-six enriched KEGG pathways were identified, including 23 pathways that showed a significant differential enrichment in the MRGBP high-expression group and 43 pathways listed in the low-expression group (Supplementary Table S1, http://links.lww.com/MD/F938). The top 9 most significantly enriched KEGG gene sets in the high-expression group were the ribosome, cell cycle, DNA replication, homologous recombination, primary immunodeficiency, Fc gamma R-mediated phagocytosis, type I diabetes mellitus, spliceosome, and leishmania infection sets, based on the NES **(**Table 4 and Fig. 3).

**Table 4:** KEGG gene sets enriched in the MRGBP high-expression phenotype.

MSigDB collection	Gene set name	setSize	NES	p.adjust	FDR
c2.cp.v7.0.symbols.gmt [Curated]	KEGG_RIBOSOME	86	2.143	0.026	0.019
	KEGG_CELL_CYCLE	124	1.99	0.026	0.019
	KEGG_DNA_REPLICATION	36	1.934	0.026	0.019
	KEGG_HOMOLOGOUS_RECOMBINATION	26	1.85	0.026	0.019
	KEGG_PRIMARY_IMMUNODEFICIENCY	35	1.827	0.031	0.023
	KEGG_FC_GAMMA_R_MEDIATED_PHAGOCYTOSIS	91	1.815	0.031	0.023
	KEGG_TYPE_I_DIABETES_MELLITUS	41	1.801	0.032	0.023
	KEGG_SPLICEOSOME	123	1.799	0.026	0.019
	KEGG_LEISHMANIA_INFECTION	70	1.799	0.031	0.023

FDR = false discovery rate, KEGG = Kyoto Encyclopedia of Genes and Genomes, MRGBP = MORF4-related gene-binding protein, MSigDB = molecular signatures database, NES = normalized enrichment score.

### 3.5 Correlation between MRGBP expression and immune infiltration

**Figure 4:**
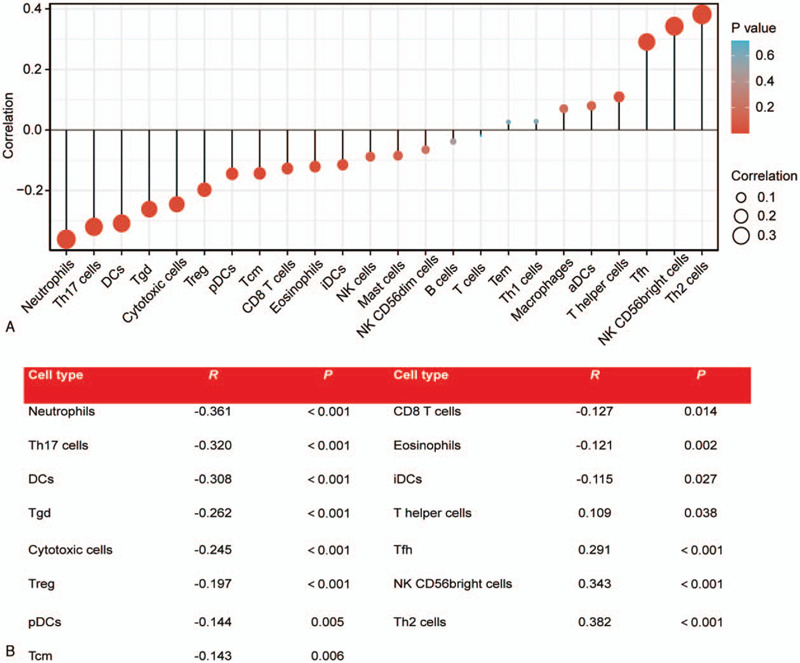
The association between the expression of MRGBP and immune cell infiltration level quantified using ssGSEA. aDCs = activated DCs, DCs = dendritic cells, iDCs = immature DCs, MRGBP = MORF4-related gene-binding protein, NKs = natural killer cells, pDCs = plasmacytoid DCs, ssGSEA = single-sample gene set enrichment analysis, Tcm = T central memory cells, Tem = T effector memory cells, Tfh = T follicular helper cells, Tgd = gamma delta T cells, Th = T helper, Tregs = regulatory T cells.

**Figure 5:**
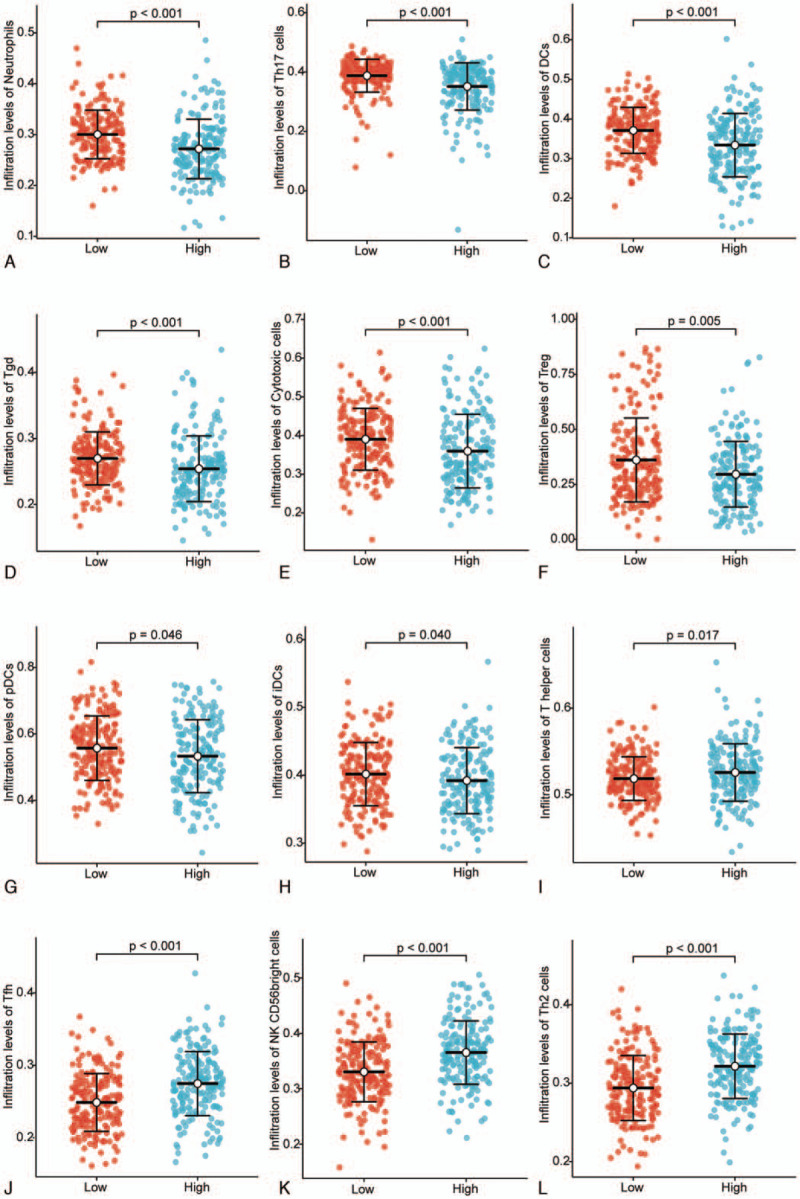
Comparison of the level of immune infiltration between high and low MRGBP expression groups in HCC. Neutrophil (A), Th17 cell (B), DC (C), Tgd (D), cytotoxic cell (E), Treg (F), pDC (G), iDC (H), T helper cell (I), Tfh (J), NK CD56 bright cell (K), and Th2 cell (L) infiltration between the high and low MRGBP expression groups. DCs = dendritic cells, HCC = hepatocellular carcinoma, iDCs = immature DCs, MRGBP = MORF4-related gene-binding protein, NKs = natural killer cells, pDCs = plasmacytoid DCs, Tfh = T follicular helper cells, Tgd = gamma delta T cells, Th = T helper cells, Tregs = regulatory T cells.

We used Spearman test to analyze the correlation between the expression of MRGBP and immune cell infiltration level, which was quantified using ssGSEA in an HCC tumor microenvironment. MRGBP expression was negatively correlated with the abundance of neutrophils, T helper (Th)17 cells, dendritic cells (DCs), gamma delta T (Tgd), cytotoxic cells, regulatory T (Treg), plasmacytoid DCs (pDCs), T central memory (Tcm) cells, CD8T cells, immature DCs (iDCs), and eosinophils. MRGBP expression was positively correlated with the abundance of T helper cells, T follicular helper cells, CD56^bright^ natural killer (NK) cells, and Th2 cells (*P* < .05) (Fig. 4). The Wilcoxon rank sum test showed that the infiltration levels of T helper cells, Tfh, CD56^bright^ NK cells, and Th2 cells in the MRGBP high-expression group were significantly higher than those in the low-expression group, and the infiltration levels of neutrophils, Th17 cells, DCs, Tgd, cytotoxic cells, Tregs, pDCs, and iDCs were significantly lower in the high MRGBP expression group (*P* < .05) (Fig. 5).

## 4 Discussion

HCC is a group of the most common primary liver carcinomas with high mortality globally. The development of treatments has tremendously improved patient survival; however, additional progress is necessary. With the advancing of immunology and molecular biology technologies such as next-generation sequencing, a variety of promising biomarkers have been identified for the early diagnosis of HCC. These include AFP, Golgi protein 73, glypican-3 (GPC-3), des-γ-carboxy prothrombin, abnormal prothrombin, heat shock protein, dickkopf-1, and osteopontin.^[18,19]^ Similarly, many molecular therapy targets have been identified^[4]^ in clinical studies. These include transforming growth factor-beta, mesenchymal-to-epithelial transition factor, fibroblast growth factor receptor 4, and GPC-3.^[20]^ However, the use of a single biomarker has limited detection capability and therapeutic efficacy. To improve the management of HCC, novel personalized and combination strategies are needed, as are further studies to reveal novel molecular targeted therapies and surveillance.^[4,19]^

MRGBP expression is frequently amplified in multiple types of cancer. MRGBP regulates cell cycle, apoptosis, tumor growth, and invasiveness. In a prior study, MRGBP expression was elevated in all 107 lung tumor tissues, and its co-expression genes were significantly enriched in signaling transduction-related pathways, such as the Ras signaling pathway, mitogen-activated protein kinase pathway, and Notch signaling pathway.^[6]^ MRGBP promotes cancer cell invasion and growth by stimulating the expression of androgen receptor target genes by promoting the recruitment of TIP60 and acetylation of a histone variant (H2A.Z) in prostate cancer.^[7,8]^ MRGBP upregulation in pancreatic ductal adenocarcinoma promotes the growth, migration, and invasion of cancer cells, suppresses apoptosis of pancreatic cancer (PanCa) cells, and has been positively associated with TNM stage, T classification, poor prognosis, and induction of epithelial–mesenchymal transition.^[10]^ MRGBP expression in PanCa cells could be directly downregulated by miR-137.^[9]^ MRGBP is also amplified in cutaneous squamous cell carcinoma, which promotes tumor growth in vivo and reduces apoptosis in vitro.^[11]^ Yamaguchi et al^[12,13]^ found that the expression of MRGBP was amplified in colorectal cancer, consistent with the findings of Carvalho et al^[14]^ that the interaction of MRGBP with bromodomain containing 8 may be key in determining MRGBP function in the proliferation of cancer cells. MRGBP can promote the proliferation of colorectal cancer cells by regulating the cell cycle, not apoptotic cells.^[13]^ However, the expression level of MRGBP in colorectal cancer was not correlated with clinicopathological factors.^[12]^ Scotto et al^[15]^ showed that MRGBP was upregulated in cervical cancer cells as a consequence of the 20q gain. Based on these studies, MRGBP may play a biological role as a diagnostic biomarker and anticancer target for tumors. However, little is known about the relationship between MRGBP and HCC. In this study, we performed a bioinformatics analysis of the prognostic value of MRGBP and the correlation between immune infiltrates and MRGBP expression in HCC.

ONCOMINE **(**www.oncomine.org**)** (the cutoffs of *P* value, fold change, and gene rank were defined as 0.05%, 1.5%, and 10%, respectively) was first used to analyze the mRNA level of MRGBP between cancer and normal tissues. The transcriptional expression of MRGBP was significantly upregulated in tumor tissues compared with that in normal tissues in 16 types of tumors (including HCC) **(**Supplementary Table S2, http://links.lww.com/MD/F939**)**. The Wilcoxon signed-rank test revealed that the expression levels of MRGBP in 371 HCC tissues were notably higher than those in 50 normal tissues. Furthermore, MRGBP expression in 50 tumor tissues was remarkably higher than that in 50 paired normal liver tissues in TCGA cohort. MRGBP expression was amplified in HCC and was significantly associated with many clinical characteristics, including T stage, residual tumor, histologic grade, TP53 status, weight, BMI, AFP, and prothrombin time. HCC with a higher MRGBP expression is more likely to progress to a poorer stage and vascular invasion than HCC with a lower MRGBP expression. Overexpression of MRGBP in HCC and its correlation with poor clinicopathologic factors indicate that MRGBP is an oncogene. Multivariate and univariate analyses demonstrated that a higher MRGBP expression indicated a shorter OS. To further study the role of MRGBP in HCC, we conducted GSEA using TCGA data. The ribosome, cell cycle, DNA replication, homologous recombination, primary immunodeficiency, Fc gamma R-mediated phagocytosis, type I diabetes mellitus, spliceosome, and leishmania infection pathways were differentially enriched in the MRGBP high-expression group. Thus, MRGBP may be a new prognostic biomarker and therapeutic target for HCC.

In addition, high MRGBP expression increased the immune infiltration levels in T helper cells, Tfh cells, NK CD56 bright cells, and Th2 cells and decreased immune infiltration in Th17 cells, DC, Tgd cells, cytotoxic cells, Tregs, pDCs, and iDCs in HCC. We infer from these findings that overexpression of MRGBP inhibits effective NK and Th1 immune responses.

The data analyzed here were retrieved from online databases, and the mRNA levels were not perfect predictors of protein expression.^[21]^ We plan to perform further cell experiments and clinical sample analyses to verify the correlation between mRNA and protein expression and the functional mechanism of MRGBP in HCC.

## 5 Conclusions

In summary, increased MRGBP expression correlates with cancer progression, poor survival, and immune infiltration levels in HCC, suggesting that MRGBP may be a novel prognostic biomarker correlated with immune infiltrates. These novel findings provide new and promising insights for subsequent research to elucidate the clinicopathological significance and molecular pathogenesis of HCC. Further experimental validation is needed to demonstrate the biological effects of MRGBP in HCC.

## Acknowledgments

I thank my son Feng Zhu. The authors also thank Editage (www.editage.cn) for the English language review.

## Author contributions

**Conceptualization:** Wei Zhu

**Funding acquisition:** Xiaoli Chen

**Investigation:** Juanjun Huang, Wei Zhu

**Methodology:** Juanjun Huang, Wei Zhu.

**Project administration:** Xiaoli Chen, Wei Zhu

**Writing – original draft:** Juanjun Huang, Wei Zhu.

All authors read and approved the final manuscript.
